# Towards a 21st century health care system: advancing the case for telecare

**DOI:** 10.1186/s13584-018-0205-4

**Published:** 2018-01-15

**Authors:** Karen B. DeSalvo, Christine Petrin

**Affiliations:** 1 University of Texas Dell Medical School, Austin, USA; 2 Tulane University School of Medicine, New Orleans, USA

**Keywords:** Telecare, Telehealth, Digital health, Value based care, Complex patients

## Abstract

Telecare is increasingly recognized as an essential tool for a contemporary twenty-first century health care system even though the evidence is still emerging on its effectiveness. The need to find delivery models like telecare that improve both the convenience and value of care is universal, but particularly pressing for countries like the U.S. and Israel who are facing rising costs related to the needs of individuals with multiple complex conditions. This commentary provides highlights of the current state of practice and policy for telecare and the challenges that remain ahead as it is adopted into the mainstream.

Telecare is increasingly recognized as an essential tool for a twenty-first century health, digital care system. Compared to even a decade ago, technology advances are making it possible to mainstream telecare with the ubiquitous nature of personal devices that support video communication, the digitization of the care experience, and a rising comfort level with digital health by clinicians and patients. Initially thought to be an opportunity to improve access to care for isolated populations, such as those living in rural settings, telecare is now being deployed as an option for the broader population.

In their article, Porath et al. add significantly to the evidence that telecare services are a cost-effective model of care. They report on services provided by the Maccabi Telecare Center (MTC), which has provided home-based telecare to over 22,000 patients, including complex patients such as frail seniors [1]. Continuous monitoring and coordinated communication allowed the MTC care team to seamlessly track frail elderly patients, incorporating multiple points of evaluation into a single integrated Electronic Medical Record (EMR), which could be viewed by patients and caregivers as well. The authors found enrollment in the MTC program resulted in significant reductions in hospitalization days and costs, as well as lower overall monthly costs for these frail elderly patients, suggesting that telecare services offer a cost-effective way to improve health outcomes in this population.

Their work builds on the existing evidence base, giving policy-makers and providers more comfort in mainstreaming telecare both financially and in practice. Previous research around the cost-effectiveness of telecare interventions has been inconclusive, and research suggests that while per-episode telecare visits are less costly than physician office visits or emergency department visits, savings from the substitution are outweighed by the increase in spending from new utilization [2]. However, for some populations, such as the frail elderly, the cost-effectiveness is clearer not only in the work of Porath, but also in U.S.-based studies [3].

The enthusiasm around the adoption of telecare lies partially in the opportunity to improve convenience for the patient and in the perceived opportunity to reduce unnecessary medical expenditures. This has led to intense interest in telecare in industrialized countries. For the U.S., cost-effectiveness is of significant concern given the high percentage of gross domestic product spent on health care and alongside poor outcomes [4]. Though Israel has a better performance record and a more affordable health care system, costs of care are rising [5]. Finding models of care that allow clinicians to practice at the highest quality and meet their patients’ needs is of concern for the rising global population of patients with complex medical needs.

Telecare programs are rapidly expanding in the U.S. with some estimates suggesting 12% of telecare visits have replaced in-person visits [2]. The remaining 88% of telecare visits represent new utilization by patients who perhaps would not typically seek care in-person, but found telecare a convenient alternative.

The system with the longest experience in telecare delivery models is the Veterans Health Administration (VHA), which is also the largest managed health care system in the United States and is run by the U.S. government. Interventions over the past 17 years have included continuous monitoring, education, mental health support and therapy, and nurse case management. A systematic review of these interventions found that seniors receiving telecare from the VHA saw improved mental health status, lower mortality rates, higher Activities of Daily Living scores, reduced likelihood of hospitalizations and primary care visits, and greater adherence and response to treatment [6, 7]. Further, these improvements were seen among elderly patients of both high and low socioeconomic status, suggesting the potential for telecare interventions to widen health disparities can be avoided [8].

Private health care systems have been increasingly active in deploying telecare with some larger systems increasingly relying on it for care access. Last year, Kaiser Permanente reported that telecare visits have surpassed in person visit [9]. Kaiser Permanente sees over 110 million physician-patient interactions annually, 52% of which occur through smartphones, videoconferencing, or other technology. Another large health system, Dignity Health, founded its telecare network in 2008, and since then has created 31 partner sites with more than 60 robots which allow specialists to virtually attend the bedside of acute care patients [10]. For patients with less acute needs, videoconferencing mediates initial informational visits as well as vital sign monitoring.

Many models are focused on improving outcomes for high-cost, high-need patient populations, particularly seniors. The IDEATel Demonstration Project used telecare networks to improve primary care to Medicare beneficiaries with diabetes. Using remote monitoring, videoconferencing, Web-based consulting and a curriculum for physicians, the demonstration improved diabetes control and lipid levels, as well as the self-efficacy of the enrollees in controlling their diabetes [11].

Outside of the hospital, an early alert system implemented by Emergency Medical Services allows the neurologic care team to evaluate patients suspected of stroke while they are still enroute to the hospital. Their continuous monitoring technology is used extensively in their asthma management program, which tracks asthma triggers and symptoms. Through the EMR online dashboard, patients can see this information and communicate with their provider about changes to their asthma management as well as receive reminders and education around proper inhaler use.

Widespread acceptance of telecare as a consumer friendly and likely cost-saving model of care has led to an increase in U.S. health insurance programs willing to pay for these services. Currently, the majority of states require equal coverage of telecare and in-person services in private insurance plans and it is estimated that all large employer plans will cover telecare services by 2020. The federal Medicare program reimburses telecare services delivered to elderly beneficiaries living in rural areas [12]. In November 2017 the Centers for Medicare and Medicaid Services announced new coverage of seven additional telecare services, including health risk assessments, psychotherapy, chronic care management, and interactive complexity [13].

Despite the uptick in deployment and the reassurance of payment for telecare services, challenges remain that prevent it from being entrenched in clinical practice. Some of these challenges will be relevant for Israel and the U.S., though Israel has health system characteristics that may allow it to outpace adoption compared to the U.S. Israel is a ripe environment due to global capitation financing, a completely digitized health care infrastructure with interoperability throughout, and no gaps in broad-band or cell access—all of which continue to challenge the U.S. health care system currently.

Understanding the potential negative consequences of telecare will be as important as understanding the value proposition. Even though there is more consumer convenience and efficiency in telecare, for physicians, care models do not yet accommodate adequate time in the clinician’s day for telecare visits or for the “set up” needed to support remote sensing devices that transmit diagnostic data essential to telecare. In the U.S., medical licensure is not granted nationally, but rather at the state level, limiting care beyond state lines. Though consumers may find the model more convenient, for the homebound, communication through video may replace some of the only human interactions they receive leading to exacerbation of social isolation and loneliness.

## Conclusion

Medical care is rapidly evolving in response to consumer demand for enhanced access, the need to reduce cost, and the opportunity to leverage technology. Telecare, though not new, is gaining acceptance because of these and other trends in health care, including innovation in consumer technology, improved EMR integration, and projected shortages in the health professional workforce. The paper by Poreth et al. provides further evidence of the benefits of telecare to improve health outcomes and reduce cost. As reliance upon telecare grows to meet the needs of a changing health care system, a stronger evidence base is urgently needed, not only to pave the way for its expansion, but also to follow its progress and build upon its successes.
